# Tuberculosis

**Published:** 1896-04

**Authors:** John Forbes

**Affiliations:** St. Joseph, Mo.


### TUBERCULOSIS.[1]

By DR. JOHN FORBES,
ST. JOSEPH, MO.

Much has been said and written recently in regard to the subject of tuberculosis, and to many it may seem a rehash to consider it again. When, however, we know that a great proportion of the deaths in the human family are due to tubercu-

[1] Read at the regular meeting of the Missouri Valley Veterinary Association, October 2, 1895.

losis, that the disease is common to man and the lower animals, and that its existence in the animals that maintain the food-supply of man is a menace to public health, and when we consider that the veterinarian must be the guardian of the public health in this respect, I think you will agree with me that it is a subject that cannot be too often considered. I regret very much that other duties have prevented me giving the attention to the subject that it demands, but what follows I trust will evoke a hearty discussion of the subject. In laying it before you for your consideration and discussion, I wish only to emphasize a few of the most important features of the subject. Koch's discovery effected a complete reformation in the clinical views of tuberculosis. All sorts of theories prevailed prior to that. Clinical medicine reconstructed itself to comply with the views imposed by the discovery.

Of the various points in the subject none seem to be more assailed than heredity. There has been no direct proof of the inheritance of the disease, but it is a clinical fact, nevertheless, and one of almost daily experience, that the disease reaps its harvest among the offspring of tuberculous parents. When the disease appears at an early age the hereditarians believe that there has been an hereditary infection instead of an early direct infection.

There are many ways in which the young can be infected. Close contact with a diseased mother or other diseased animals, and the contamination of the food with sputum containing the bacilli. Milk, however, especially in the human subject, is the most frequent source of infection, and it is surprising how little the general public are enlightened upon this subject. They recognize that the disease exists in the beef-supply, and they consider a rigid inspection of meat a matter of necessity, but they seldom think of the dangers lurking in one of the most universal articles of diet. Even in intelligent families too little attention is paid to the source of the milk-supply for children. It may be that the fear of infection by milk is exaggerated, but one can hardly think so when we hear of the frequency of tuberculosis of the mesenteric glands in the young. It is obvious, then, that a great responsibility rests upon those who have the supervision of the health of children.

There are other methods by which infection may take place and which may simulate hereditary transmission. A diseased progenitor may contaminate the stables or utensils and impart the disease to the young, and the less space allowed to animals and the closer they are confined increase the danger.

While we cannot believe in hereditary transmission, experience shows that the offspring of tuberculous progenitors sooner or later manifest the disease when exposed to the exciting cause, and we also know that nurses and attendants in consumptive hospitals, while exposed continually to the danger of infection, very infrequently contract the disease. We are thus compelled to acknowledge that a healthy body can resist the attack of the bacillus, and that the bacillus in order to flourish must have a suitable pabulum. Vigorous parents will bestow upon their offspring a vigorous constitution, and weakly parents bestow upon their offspring their habits of body. In this bestowal of a weakly habit of body on the offspring we may find an explanation of the theory of heredity. But it matters not if we do have this weakly constitution, the bacillus is an absolute necessity to the propagation of the disease. The bacillus of tuberculosis is not ubiquitous. It only exists in the immediate vicinity of a tuberculous patient, hence it must exist plentifully in the popular resorts for consumptives, and we must conclude that when the resorts are well patronized by tuberculous people they are dangerous places for delicate persons.

Besides this inherited weakness of the system, the so-called "tubercular diathesis," there are other influences which may reduce the organism to the state suitable for the propagation of the bacillus. In cattle the most potent influences are overcrowding and excessive milking, a very common occurrence in city dairies, and let me impress upon you the fact that a phthisical attendant of dairy cattle may be a source of danger, not only to the health of the cattle, but to the public who consume the product of that particular dairy. The city that does not have a scientific supervision of the source of its milk-supply lacks in its duty to its citizens. There are cases on record of a phthisical patient who imparted the disease to chickens in his yard that devoured the sputa; of another who inflicted the disease upon two pet dogs in succession, and would have given it to the third had the patient not died before it could be affected. If that can be said of these animals, might not the same be true in regard to cattle? Even if it were not, there is always the danger of the milk becoming infected through the dried sputa of the diseased attendant, and we all know that milk is one of the most frequent habitats of diseased germs.

We have seen that the bacillus must find entrance to the body before we can have tuberculosis, and it may be of interest to us to know how it gains access. Infection through wounds has often been recorded, and in such cases the process of repair is retarded, the infective process spreads to the adjacent glands, and ultimately results in general tuberculosis. Fraenkel relates that this has taken place in surgical operations. Through the alimentary canal infection often takes place by the ingestion of contaminated food. Being able to resist the action of the gastric juice, the bacilli readily gain entrance to the abdominal glands. The most frequent point of entrance of the bacillus is the respiratory tract. As with the other methods of infection, the same conditions do not prevail in this instance. The substance that contains the bacilli must first dry up and be reduced to a powder, and the bacilli be lifted into the air, from which it is inhaled. Only such bacilli as withstand the drying process can enter this way, and it must be remembered that the tubercle bacilli has a great power of withstanding this drying process. This theory as against the other, that the bacteria free themselves from where they are contained and are carried upward by the air or by evaporation, has been clearly proven by a series of beautiful experiments related by Fraenkel in his book on bacteriology. Dust collected from a dwelling where dwelt a phthisical patient produced tuberculosis in a guinea-pig. Dust taken from a place where there was no consumptive produced negative results. It was found that the dried sputa found in the pocket-handkerchiefs of consumptives was a prolific source of the bacteria. As I have already indicated, every consumptive is a source of danger to the health of animals as well as to the people around him.

The bacilli having gained access to the body, by virtue of their biological quality, they originate an irritation resulting in a true inflammation. It is then that a struggle for supremacy takes place between the cells of the tissue on the one hand and the bacilli on the other, and the result is determined by the vitality of the cells or the infectiousness of the bacilli. Should the cells conquer the bacilli are destroyed before they can do any injury, but if the bacilli conquer the result is well known.

It may interest us to glance at the theory of phagocytosis. Phagocytes are formed of leucocytes and fixed connective-tissue cells. They are of two kinds, the large and the small. It seems to be the duty of these elements to fight the battle on behalf of the organism against the invasion of the bacilli. As soon as the irritation is set up they hasten to the battlefield, and the fight is on to the death. The phagocytes endeavor to devour the invaders, to digest them, and render them sterile. It seems that certain bacilli do not require the whole force of the phagocytes. For instance, the streptococcus of erysipelas can be overcome easily by the small phagocytes, as can other micrococci; but in the case of tubercle bacilli both the large and the small phagocytes take part in the fray, evidently proving that the bacillus tuberculosis is a formidable adversary.

I bring this theory to your notice in the hope that we can elicit some discussion on the subject, as I have never heard the question considered from this point, and I hope to gain some information from it.

---

A 1½ to 2 per cent. watery solution of trikresol will be found of great value in mild cases of eczema and in the early stages of mange of dogs; it is also useful where rubbing and itching bases of tails prevail among colts or horses when wintering.

The use of injections of solutions of carbolic acid as a preventive of infectious abortion among cattle should be given more extensive trial and investigation.

The better care of nail-wounds among horses in Delaware has resulted in a lowered death-rate from tetanus. The favorable use of tetanin in one case is reported, though the case ran the usual course.

Ringworm in cattle, especially among calves, may be dealt with successfully by using corrosive-sublimate washes, followed by painting with tincture of iodine.

The latest researches establish only indirect relationship between the large bloodvessels in the region of the udder and the quantity and quality of the milk-production. Further, that milk is an original secretion, similar to the product of the salivary gland, and not merely changed blood. Its ease of flow and fluctuations in quality are the results of disturbances, such as pain, inflammation, worriment, which exert their direct influences on the nerve-supply of the mammary glands.

